# Redundancy Reduction for Sensor Deployment in Prosthetic Socket: A Case Study

**DOI:** 10.3390/s22093103

**Published:** 2022-04-19

**Authors:** Wenyao Zhu, Yizhi Chen, Siu-Teing Ko, Zhonghai Lu

**Affiliations:** 1 School of Electrical Engineering and Computer Science, KTH Royal Institute of Technology, 10044 Stockholm, Sweden; wenyao@kth.se (W.Z.); yizhic@kth.se (Y.C.); 2 Research and Innovation, Össur, 110 Reykjavík, Iceland; stko@ossur.com

**Keywords:** pressure sensor system, prosthetic socket, redundancy detection, redundancy reduction, selforganizing map, Pearson correlation coefficient

## Abstract

The irregular pressure exerted by a prosthetic socket over the residual limb is one of the major factors that cause the discomfort of amputees using artificial limbs. By deploying the wearable sensors inside the socket, the interfacial pressure distribution can be studied to find the active regions and rectify the socket design. In this case study, a clustering-based analysis method is presented to evaluate the density and layout of these sensors, which aims to reduce the local redundancy of the sensor deployment. In particular, a Self-Organizing Map (SOM) and K-means algorithm are employed to find the clustering results of the sensor data, taking the pressure measurement of a predefined sensor placement as the input. Then, one suitable clustering result is selected to detect the layout redundancy from the input area. After that, the Pearson correlation coefficient (PCC) is used as a similarity metric to guide the removal of redundant sensors and generate a new sparser layout. The Jenson–Shannon Divergence (JSD) and the mean pressure are applied as posterior validation metrics that compare the pressure features before and after sensor removal. A case study of a clinical trial with two sensor strips is used to prove the utility of the clustering-based analysis method. The sensors on the posterior and medial regions are suggested to be reduced, and the main pressure features are kept. The proposed method can help sensor designers optimize sensor configurations for intra-socket measurements and thus assist the prosthetists in improving the socket fitting.

## 1. Introduction

As the interface between the amputation stump and prosthesis, the prosthetic socket is the key factor that affects the comfort level of patients. However, as the cause of amputation and residual limb characteristics vary from patient to patient, the design and adjustment of the socket shape remain a hard problem. Normally, the rectification of socket shape has to consider physical conditions such as the pressure, shear stress and residual limb volume fluctuations [1,2] during activities of daily living. The measurement and analysis of these interface pressure loading conditions then become a key component in optimizing the socket shape and improving the comfort level of patients.

**Figure 1:**
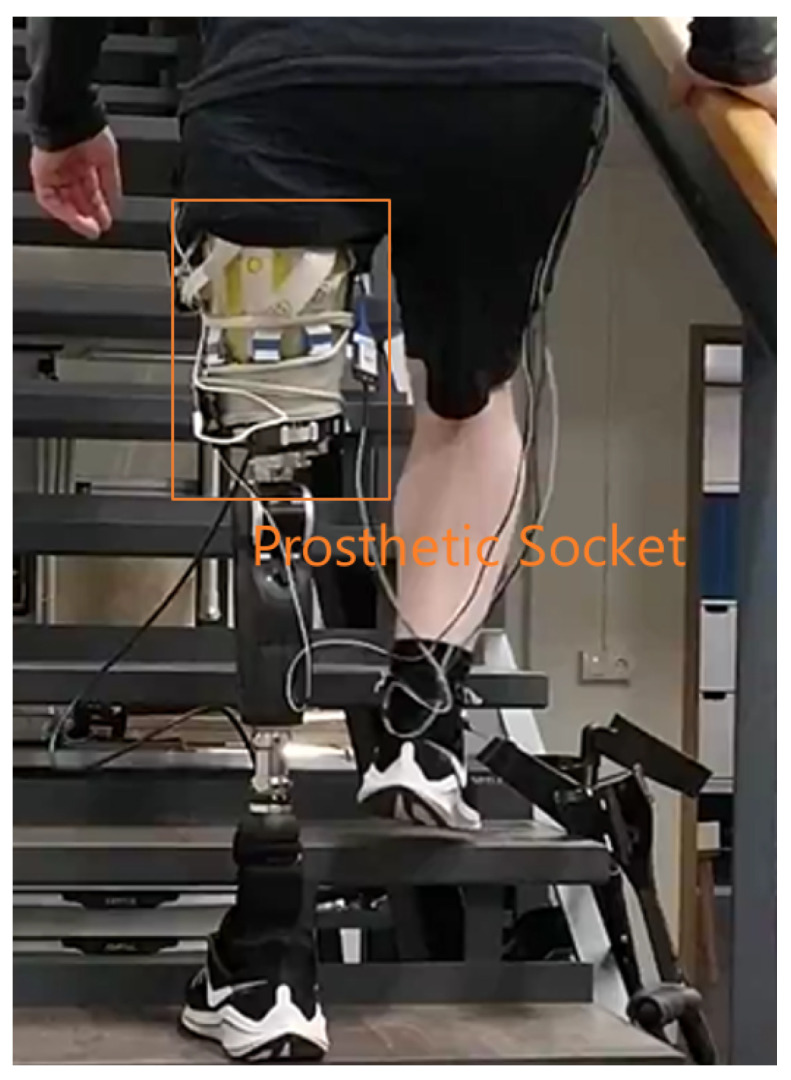
Amputee patient wears a prosthetic socket.

Uneven and high interfacial pressure distribution of the prosthetic socket can lead to many discomforts in amputees’ daily lives [3], while the development of the sensing technology [4] helps identify such stresses. Figure 1 shows an amputee patient who wears a prosthetic socket with the sensor measurement system in a clinical test. Such sensor systems usually have high density [5,6,7,8] to ensure the coverage of the whole stump. The high-density coverage may, however, introduce redundancy, which increases cost and unnecessary complexity. Therefore, detecting and removing the dispensable sensors while keeping the effectiveness of the pressure measurement becomes crucial in pressure studies of prosthetic sockets.

This research intends to reduce the local redundancy in the sensor deployment by proposing a clustering-based analysis method that can locate the unnecessary sensors by evaluating the data from a predefined sensor layout and then provide suggestions to lower the current sensor density. The clustering method, such as a Self-Organizing Map (SOM) [9], is employed to find the clustering results of the sensor data. The most common one is selected as the redundancy detection model for the sensory system. Then, the similarity metrics, such as the Pearson correlation coefficient (PCC) [10], are applied to indicate the unessential sensors in the relevant regions and evaluate the efficacy of the modification on the sensor layout. Jenson–Shannon Divergence (JSD) [11] is applied as a validation metric from the pressure distribution perspective to check if the new sensor layout is credible. Overall, the presented method aims to improve the sensor deployment no matter what kind of pressure sensor or which individual amputee is being treated.

Our redundancy reduction method can select the most effective positions in which pressure sensors are integrated. This has the potential to be employed in smart socket design, for example, in a closed control loop of an actuation system for efficient data acquisition with reduced weight and complexity of the final system.

The rest of the paper is organized as follows. In Section 2, the related works on sensory systems for the prosthetic socket are introduced. Section 3 defines the sensor deployment problem in the interfacial pressure study. In Section 4, the clustering-based analysis method and the related algorithms are explained. In Section 5, the experimental results of the study case and discussion are presented. In Section 6, we draw the conclusion and discuss the future works.

## 2. Related Works

The measurement of the interfacial pressure is critical to understanding the comfort level of patients in the prosthetic socket design. Several researchers have performed physical testing on the socket in the prosthetic study. Commuri et al. [12] have assessed the pressure distribution during walking inside the transfemoral socket with triaxial force sensors mounted on the socket wall. Tran et al. [13] analyzed the pressure and the shear stresses of an amputee with the sensors embedded in the socket and visualized the pressure map according to the sensor readings.

The clinical trial from Ali et al. investigated the interfacial pressure during walking with hundreds of resistive transducers placed on the residual limbs [6], and they also studied the pressure during the stair ascent and descent activities using the same sensory system [7]. Although these researchers used a large number of sensors during the test, their analysis normally focused on the mean peak pressure, the standard deviation and other statistical features of the pressure in four major areas, such as anterior, medial, posterior and lateral. The results in the sub-regions under these major areas are quite similar, which indicates the redundancy in the sensor layout. This implies that some of the sensors can be removed inside these regions, and the important features of pressure distribution are not lost. When reducing the sensor density, the time and cost of the measurement can also be decreased.

There have been attempts to reduce the sensor density when the researchers are building their own sensing system. Jasni et al. [8] developed the in-socket sensory system based on the analysis of Tekscan F-Socket sensors. Their method was to locate the sensors in the most active muscle areas determined by a muscle assessment of a particular amputee. However, they did not investigate the redundancy that existed in sensor placement inside the socket as their research was not focused on solving the sensor redundancy problem.

The fuzzy clustering method has been used for the redundancy detection in the environment monitoring aspect, where the air pollution data were collected monthly from all over the world [14]. Their analysis method is not suitable for intra-socket measurements since the sensors inside the socket have much higher density and frequency. The premise of clustering, however, can reveal the similarity in the sensor readings and thus detect the redundancy in sensor layout. In our case, the Self-Organizing Map (SOM) is a good choice as it can preserve the topology information on the 2D grid [9]. Compared with the K-means algorithm [15] and fuzzy c-means clustering algorithm [16], SOM allows empty clusters in the output and one sensor can only be placed in one cluster. To select the essential sensors inside the socket according to the clusters, the SOM algorithm provides more stable clustering results for redundancy detection.

Our approach can be potentially applied in smart socket design to achieve efficiency and reduce complexity. In the field of smart socket design, Paternò et al. [2] quantified the residual limb volume fluctuations in transfemoral amputees using a 3D optical scanner. An app-controlled motor-driven adjustable socket [17] and a layer jamming actuator-driven shape-changing socket [18] were developed to improve the management of the changes in patients’ limb volume. Weathersby et al. [19] implemented a motor-actuated, cable-panel socket that automatically maintains a socket fit based on distance information collected by an inductive sensor embedded in the socket wall.

Our work proposes an effective method to reduce sensor layout density for interfacial pressure measurement in the prosthetic socket. Specifically, we use a clustering method to detect redundancy, a similarity-based method to guide the removal of non-essential sensors, and use a distribution similarity metric to validate that the redundancy removal results in acceptable information loss.

## 3. The Wearable Sensor Deployment Problem in Prosthetic Sockets

**Figure 2:**
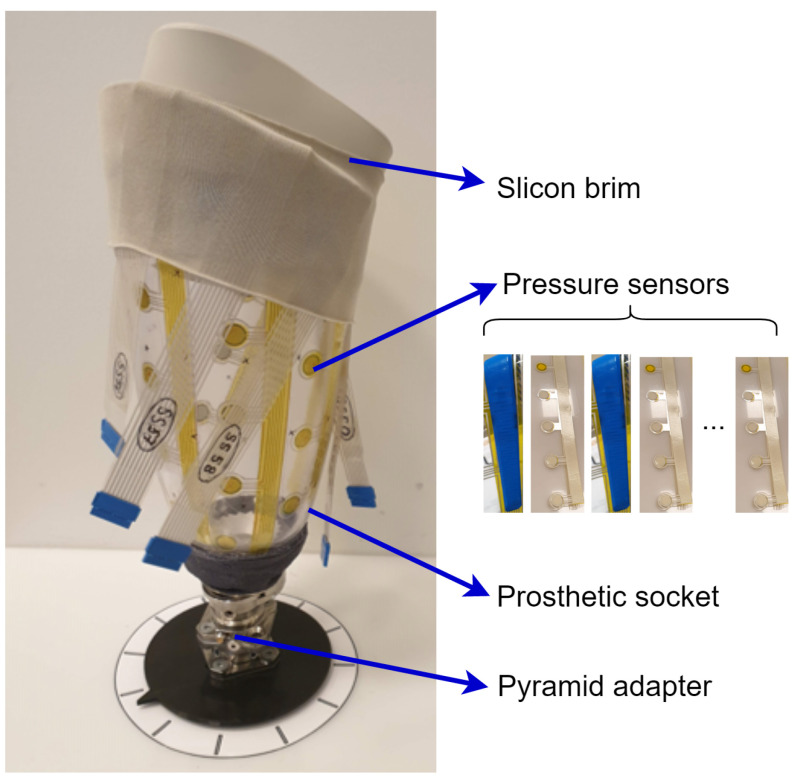
Prosthetic check socket with sensors attached.

The wearable sensors for interfacial pressure measurement are made flexible and rather thin so they can be easily mounted in prosthetic sockets. Figure 2 gives an example of the wearable sensor system placed in a transfemoral socket, where the sensors are fixed by adhesive tapes. Usually, the researchers use hundreds of sensors to reach high coverage on the surface of the whole stump. However, handling those sensors in the clinical test for one subject can take a few hours with many types of measurement equipment attached to provide enough channels for sensor reading. These tests may continue with the fitting of the socket shape for several iterations, which is a time-consuming activity [20].

Hence, the sensor deployment appears to be a challenging problem as a trade-off between the measuring coverage and the efficiency. The efficiency is improved by using a smaller number of sensors and less time in clinical trials with an acceptable loss in interfacial pressure measurement.

Since large redundancy exists in the pressure data analysis process, the pressure distribution is quite uniform in some of the areas with less muscle contraction [8]. This also implies redundancy in the data acquisition process. How to detect and remove the redundancy of the sensor placement during the data collection then becomes a key point to this problem. A redundancy reduction method is required to deal with it and to reduce the sensor density by removing the unessential sensors.

After the removal of the sensors inside the socket, it is necessary to validate that similar pressure features such as the distribution and mean pressure can still be obtained with the optimized sensor deployment.

In this paper, we present a case study with a focus on reducing sensor redundancy to optimize the sensor deployment for interfacial pressure measurement for prosthetic sockets. The details of our method are described in Section 4.

## 4. Sensor Redundancy Reduction Method

### 4.1. Overview

To optimize the sensor deployment for the socket interfacial pressure measurement, a key factor is to reduce the redundancy in the sensor layout. Since there has been extraordinary variability in the amputee situation and prosthetic socket configuration, there are no common sensor deploying rules. We proposed a clustering-based method to detect and remove the sensor redundancy for interfacial pressure measurement for amputees, and it could be general guidance for socket sensor deployment.

Based on the practical experience of prosthetists, a preliminary sensor layout is established for the general cases. Then, an intra-socket measurement with this layout on a real patient is required to obtain the initial pressure readings. According to the pressure data, we aim to find the similarity between them since the redundant sensors would generate correlated pressure curves when they are adjacent to each other. Intuitively the unsupervised clustering methods can be employed for this task, which separates the sensors into groups according to their similarities and indicate the existence of unnecessary sensors in the local region.

**Figure 3:**
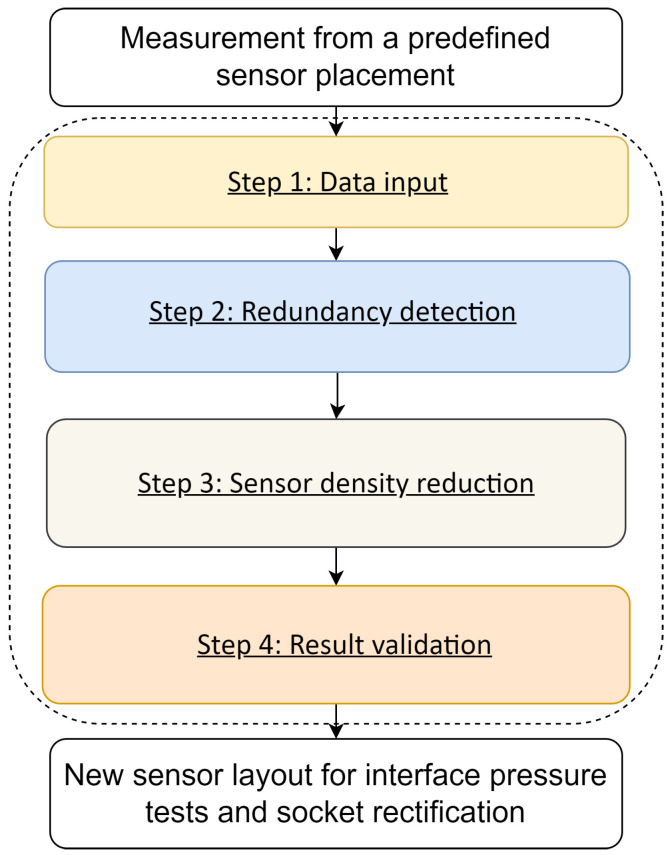
The clustering-based analysis method process.

Figure 3 shows the overall process of the clustering-based analysis method for reducing redundant sensors, which consists of four functional steps to generate a new sensor deployment scheme.

1. **Data input:** The sensor data may have various formats depending on the sensory system, and they need to be converted to the actual pressure in the same unit. After that, the pressure data are cleaned and sliced into frames, which contain several gait cycles, to maintain enough information during the dynamic tests. These frames constitute the initial dataset for the analysis method.
2. **Redundancy detection:** By adjusting the parameters of the SOM and feeding with different data frames from the initial dataset, multiple clustering results are learned. Among them, one common clustering result (which appears the most for all the data frames and model configurations) is picked up as the target model to detect the sensor redundancy for the input case.
3. **Sensor density reduction:** According to the redundancy detection model, the local redundancy in current placement is recognized. Considering the actual requirements and the capability of the sensory system, the unnecessary sensors can be removed from the corresponding clusters. The similarity metrics such as PCC can be used to guide the selection.
4. **Result validation:** The sensor removal results need to be evaluated based on the choice from step 3. The pressure distribution over the whole test from the initial sensor layout will be compared with readings from the reserved sensors, using entropy-based metrics such as the Jenson–Shannon Divergence (JSD). After the posterior evaluation, we can determine how dependable our sensor selection is.

The last step of the proposed method would provide a new sensor layout for the input case with a pruned layout. If the number of sensors to be maintained changes because of the test conditions and the patient situation known from the prosthetist, we return to step 3 and go through step 4 again to obtain another sensor removal suggestion.

**Figure 4:**
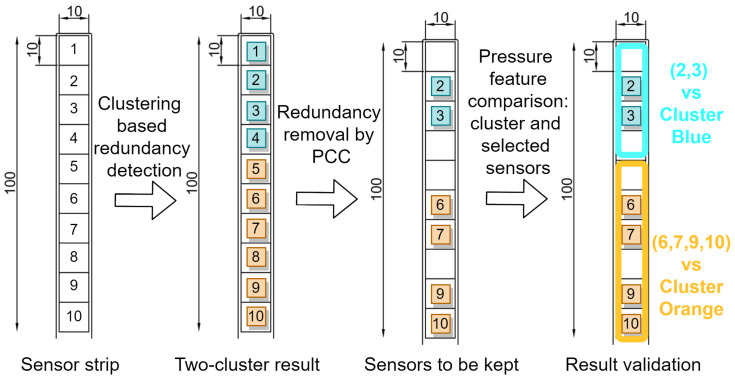
An example of a redundancy removal procedure on a sensor strip consisting of 10 sensors.

Figure 4 provides an example of the redundancy removal process of a dedicated sensor deployment with our method. The original layout is a long strip with ten sensors on it, which are labeled with numbers 1 to 10. By applying the clustering method, we can separate them into two clusters, sensors 1 to 4 are grouped in the blue cluster, and sensors 5 to 10 are grouped in the orange one. Suppose the prosthetist would like to keep two sensors for the blue cluster and four for the orange cluster based on the redundancy detection model, then the similarity metric is checked within the two clusters. Finally, sensors 2, 3 from the blue cluster and sensors 6, 7, 9, 10 from the orange cluster are reserved to form the new layout. Finally, validation metrics such as JSD and mean pressure are used to compare the information loss between the cluster and the selected sensors. The new sensor placement can be applied for future tests.

### 4.2. Redundancy Detection and Clustering Algorithms

The second step in our method uses the clustering algorithm to build the sensor redundancy detection model. Here, we will introduce the Self-Organizing Maps (SOM) [9] as the main method for generating the clustering results.

SOM is a two-layer neural network model that contains the input layer and competitive layer. The number of neurons in the competitive layer represents the number of clusters for output. Suppose we have an input set of time series $x_{i}$, with the length of *n*, and the weights connect the two layers between the *i*th input neuron and the *j*th output neuron $w_{ij},i=1,\cdots ,n,j=1,\cdots ,k$. *k* is the number of expected clusters. τ is the total iteration number. Then, the learning process can be described as follows:

1. **Initialization:** The weights of the SOM are first initialized, e.g., with some small random numbers.
2. **Competition:** Each input will find its best matching unit using some judgement methods in a time series, i.e., the distance metric. The winning unit is called the best matching unit (BMU).
3. **Cooperation:** The BMU decides the range of its neighbors to update weights. Suppose we use the Gaussian distribution, $\sigma \left(t\right)=\sigma_{0}\exp\left(-\frac{t}{\tau }\right)$ is the parameter decay as iteration grows, with the initial standard deviation $\sigma_{0}$ and current iteration number *t*. Then, the update distribution *T* of node *j* is given by $T_{j,BMU}\left(t\right)=\exp\left(-\frac{D^{2}}{2\sigma {\left(t\right)}^{2}}\right),$ and *D* is the geometry distance between node *j* and BMU. The closer neighbor will obtain a larger update.
4. **Adaptation:** The weights of the neurons are updated by $\Delta w_{ij}=\eta \left(t\right)\times T_{j,BMU}\left(t\right)\times \left(x_{i}-w_{ij}\right).$ In the equation, the learning rate is defined as $\eta \left(t\right)=\eta_{0}\exp\left(-\frac{t}{\tau }\right).$
5. **Iteration:** Go back to the above steps from the competition until τ iterations are complete. The final winning neurons of the input time series are their clusters.

The SOM uses a kind of global decision-making strategy, which could avoid falling into the local minimum. It can still be stable and accurate when the noise data exists. On the other hand, it relies on the learning parameters and can generate different clustering results on the same dataset. Hence, we have to train the SOM on multiple data frames sliced from the original dataset and treat the most common clustering result as the final redundancy detection model on all frames.

In addition, we can use other clustering methods such as the classic K-means algorithm to perform the cross-validation. The K-means algorithm aims to minimize the distance between all input vectors to the corresponding centroids based on the least-squares method [15]. It will be used on the same data frames to see if there is a common one among all the frames. We compare it with the SOM result to evaluate the reliability of the redundancy detection model.

### 4.3. Metrics for Guiding Sensor Removal

The Pearson correlation coefficient (PCC) is one of the most popular similarity metrics used by experimental researchers [21]. The equation of PCC [22] between two series is calculated as (1). The $X_{i}$ and $Y_{i}$ are the *i*th data points on time series *X* and *Y*. $\bar{X}$ and $\bar{Y}$ are the average values of *X* and *Y*.

(1) $$\mathrm{PCC}\left(X,Y\right)=\frac{\sum_{i=1}^{n}\left(X_{i}-\bar{X}\right)\left(Y_{i}-\bar{Y}\right)}{\sqrt{\sum_{i=1}^{n}{\left(X_{i}-\bar{X}\right)}^{2}}\sqrt{\sum_{i=1}^{n}{\left(Y_{i}-\bar{Y}\right)}^{2}}}$$

PCC is a measure of linear correlation between *X* and *Y* and it ranges from −1 to 1. A high PCC close to 1 shows a linear relationship between *X* and *Y* with a positive slope, which implies a similar trend of *X* and *Y*.

**Figure 5:**
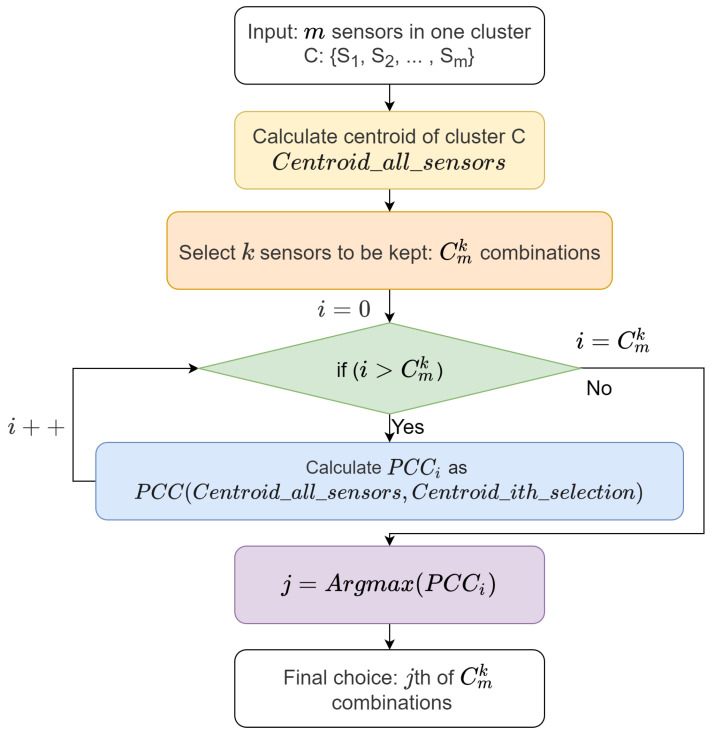
PCC-based sensor removal procedure.

Based on PCC, we can suggest the removal of unessential sensors. Suppose we have a sensor dataset and the related redundancy detection model. Figure 5 shows the sensor removal procedure. For a cluster containing *m* sensors, the mean values of all sensor data in that cluster are calculated as centroid_all_sensors. If we select *k* sensors to be kept, there are $C_{m}^{k}$ combinations. *k* should be a value between 1 and *m*, and it is flexibly set based on the knowledge of prosthetists. The mean value of the *i*th combination of sensors to be kept in that cluster is computed as centroid_ith_selection. We then calculate the PCC for each pair of centroid_all_sensors and centroid_ith_selection. The selection with higher PCC is better. Assuming the *j*th combination has the highest PCC, we use it as the final choice.

For one specific number *k*, our proposed method selects *k* sensors to be kept and remove other sensors. Our proposed approach provides flexibility for prosthetists to select the number of sensors to be kept, as the method possesses the scalability to find the *k* sensors to be kept without a limitation on the value of *k*.

### 4.4. Validation after Sensor Removal

To validate whether the pressure readings after the removal of redundant sensors can still represent those from the initial sensor layout, we need to perform a posterior check based on the selected sensors. As the pressure distribution among gait cycles is an important factor for socket fitting [4], it can be used as a reference for validation. For each cluster we obtain from the redundancy detection model, a pressure distribution *P* can be obtained on the whole input dataset from all sensors in this cluster. On the other hand, for the selected sensors in that cluster, they will constitute another pressure distribution *Q* over the same walking phase. Then, the difference between these two distributions can be found by the statistical divergence.

In our case, since the *P* contains the information of *Q* and they are in the same cluster, they should have enough overlapping, then the Jenson–Shannon Divergence (JSD) [11] can be a suitable similarity metric between *P* and *Q*. JSD is a bounded symmetry metric based on the Kullback–Leibler Divergence (KL) [23], which means JSD(P||Q)=JSD(Q||P) with a value between 0 and 1. When JSD(P||Q) close to 0, *P* is similar to *Q*. The calculation of JSD is given in (2), in which p(x) and q(x) are probabilities from distribution *P* and *Q*.

(2) $$\begin{array}{cc} KL(P||Q) & =\sum p\left(x\right)\log\frac{p(x)}{q(x)},\\ M & =\frac{1}{2}\left(P+Q\right),\\ JSD(P||Q) & =\frac{1}{2}KL\left(P||M\right)+\frac{1}{2}KL\left(Q||M\right). \end{array}$$

By examining the possible combinations of the selected sensors, we can have multiple pressure distribution *Q*s. Then, we calculate the corresponding *JSD*s and evaluate if the selection has good coverage of the pressure distribution compared with the original sensor layout. The selections with lower JSD can present the information from all the sensors better, and we use this metric as a validation of our approach. In addition to the pressure distribution, the mean pressure during walking is also an important feature for the prosthetist to investigate [7]. It is also included as a validation metric.

## 5. Experiments and Results

### 5.1. Sensor Data Acquisition

To show the efficacy of the presented analysis method, we conducted a case study. A set of pressure data is acquired from a clinical trial on a transfemoral amputation patient. The clinical investigation was conducted in compliance with related regulations and guidelines and in accordance with the ethical principles that have their origin in the Declaration of Helsinki.

**Figure 6:**
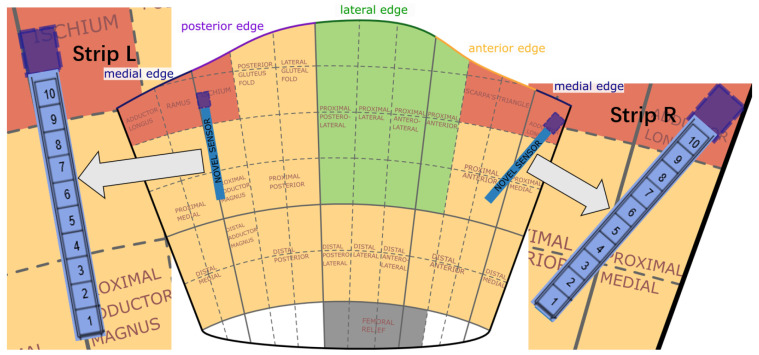
A 2D representation of a prosthetic socket with Novel [27] sensor placement (2 strips with 10 sensors on each strip) inside the socket.

The socket used in the experiment is manufactured for the user according to a scanned positive mold of the subject’s residual limb. The main material of the socket is transparent Polyethylene Terephthalate Glycol (PETG plastic). It is a replica of the patient’s own direct socket [24] manufactured by Össur with a soft silicone brim. Figure 6 draws the 2D coordinate map of the socket according to the shape of the socket. The research from Neumann et al. [25] obtained a dynamic socket pressure mapping in a suitable socket, which indicates the windows for pressure-sensitive areas. The regions divided by different colors in Figure 6 refer to their hypotheses, where red indicates high pressure and green indicates low pressure on average. The blue strip indicates the sensor position along the posterior edge (strip *L*) and the medial edge (strip *R*). The measurement device to read pressure data from sensors is Pliance [26] from Novel Electronics Inc., and two sensor strips used in the test are Novel S2006 [27], with 10 capacitive pressure sensors on each strip (Single sensor area: 10×10 mm^2^, Sensor pressure range: 2–200 KPa, Thickness < 1.2 mm, Pliance reading range setting: 0–64 KPa, Pliance reading frequency setting: 100 Hz).

The conducted test is indoors at the ground level walking at the patient’s self-selected speed. The first 100 steps are for the sensor calibration and are excluded from the test results. Then the pressure is recorded at a rate of 100 Hz during the rest of the test.

To build the dataset, we take 30 s off the pressure readings from both of the positions, namely dataset *L* for the posterior region and *R* for the medial region. Each of the datasets contains ten sensors and with a length of 3000 points.

**Figure 7:**
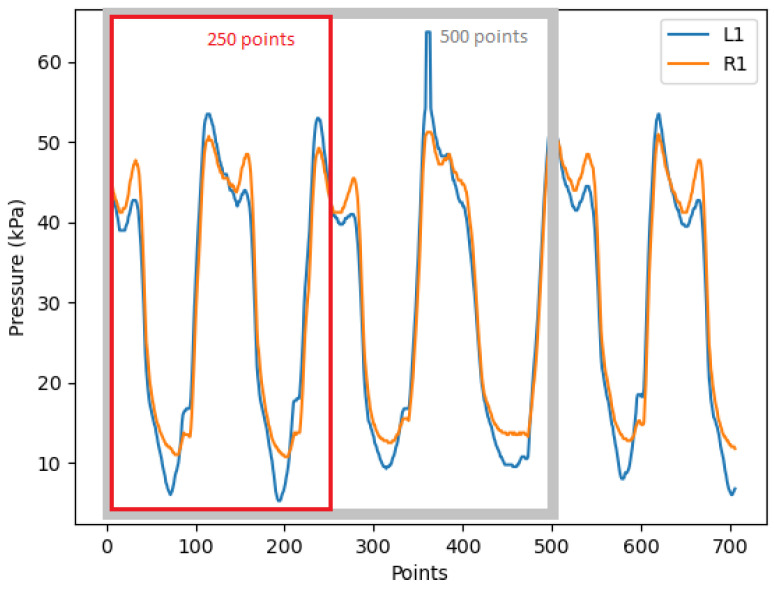
A sensor data frame in lengths of 250 and 500 points. (L1 is the first sensor in dataset *L*, and R1 is the first sensor in dataset *R*).

After that, we slice the dataset into multiple frames of shorter length. The frame length has an influence on redundancy detection as it contains a different number of gaits. In Figure 7, the trough to trough is equivalent to one full gait cycle. For example, 250 data points comprise two gait cycles and 500 data points contain around four gait cycles, which are framed by the red and grey rectangle, respectively. Other researchers like Jasni et al. [8] have used a frame size of five seconds for in-socket pressure tests on transfemoral amputees. In our experiment, we also choose 500 data points (5 s) as the frame length as it includes four to five gait cycles depending on the patient’s speed, which can provide enough information on the pressure changes during walking. We will also test the frame length of 250 points and compare it with the redundancy detection result of 500 points.

### 5.2. Redundancy Detection

The clustering methods are applied to the six frames, each with a length of 500 points, from the dataset *L* and *R*, respectively, to build the redundancy detection models. For the SOM, we set the output layer as a 3×3 rectangular grid, as the number of input entries is ten. The learning rate is set to 0.2, and the SOM runs for 10 iterations. Then, six clustering results would be generated for each of the datasets, and the most common one will be selected as the final clustering result.

**Figure 8:**
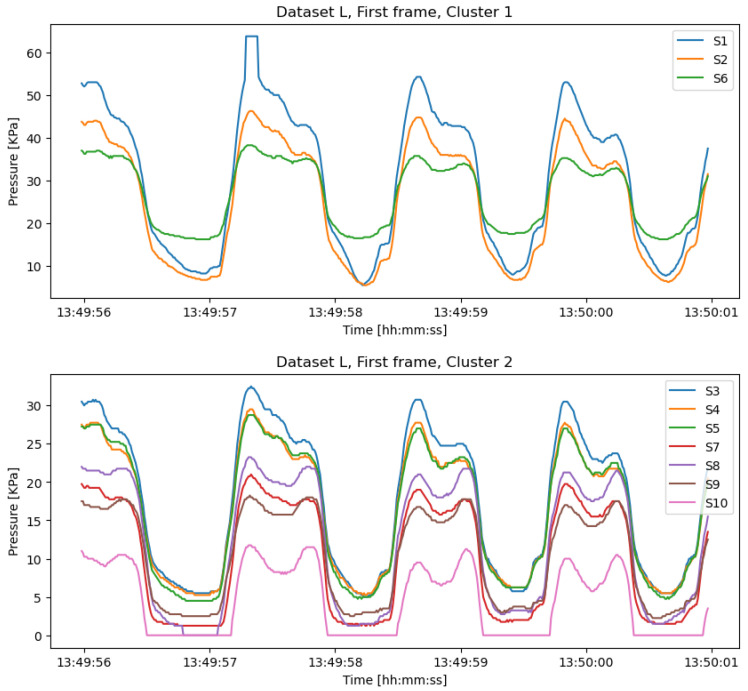
Cluster1 and Cluster2 of the first data frame from dataset *L* in the posterior region.

Figure 8 plots the clustering result for the first frame on dataset *L*, which has two non-empty clusters. Sensor numbers 1, 2, and 6 are in the same cluster, while the other seven sensors are in the second cluster. Actually, for six frames of *L*, we only obtain one different clustering result on the fifth frame, which is {1,2,3,6} and {4,5,7,8,9,10}. The other five frames are clustered by $C_{l_{a}}$:{1,2,6} and $C_{l_{b}}$:{3,4,5,7,8,9,10}. Therefore, we consider this two-cluster result as the redundancy detection model $M_{L}\left(C_{l_{a}},C_{l_{b}}\right)$ for *L*.

Similarly, the SOM algorithm gives the clustering results $C_{r_{a}}$:{1,2,4,5,6} and $C_{r_{b}}$:{3,7,8,9,10} for all six data frames in the medial region dataset *R*. The consistency of the clustering result proves that the way to divide sensors in such groups is reasonable. Then, we can determine the redundancy detection model $M_{R}\left(C_{r_{a}},C_{r_{b}}\right)$. Finally, we have $M_{L}$ and $M_{R}$ for the sensor removal steps on datasets *L* and *R*, respectively.

Considering the same dataset with 12 frames, each with length of 250 points. With the same parameters for the SOM, the most common clustering result of *L* is $C_{l_{a}}$:{1,2,6}, $C_{l_{b}}$:{3,4,5} and $C_{l_{c}}$:{7,8,9,10}, in which 7 out of 12 frames are the same. The most common clustering result of *R* is $C_{r_{a}}$:{1,2,4,5,6} and $C_{r_{b}}$:{3,7,8,9,10}, in which 4 out of 12 frames are the same, while some of the frames even have four non-empty clusters after clustering. Although the final clustering results of this test are quite close to the $M_{L}$ and $M_{R}$ we obtained from the 500-point frame experiment, we can see that the clustering results are quite unstable among different frames from the same strip. Therefore, the smaller frame size can lead to unstable clustering results, and 500 points per frame is a good choice to obtain a relatively consistent result over the whole dataset.

In addition, we use the K-means on all six frames in lengths of 500 points on the same dataset for *L* and *R*. Since we have the two-cluster model from SOM, here we set *K* to 2 and run the algorithm for 10 iterations as well. The SOM model on dataset *L*, $M_{L}:C_{l_{a}}$:{1,2,6} and $C_{l_{b}}$:{3,4,5,7,8,9,10}, appears three times out of the six frames in K-means clustering. The SOM model on dataset *R*, $M_{R}:C_{r_{a}}$:{1,2,4,5,6} and $C_{r_{b}}$:{3,7,8,9,10}, also appears three times out of six in K-means clustering. Though the K-means model only has half the number of frames in both datasets with the common clustering results, they contain the same clusters as the SOM results. Therefore, the redundancy detection model on these two datasets can be determined as $M_{L}$ and $M_{R}$, respectively.

### 5.3. Sensor Removal Result

In this section, we present the results of choosing one sensor or two sensors, while the approach we proposed has the flexibility on the number of sensors to be kept. It is not limited to one or two. Our method aims to provide suggestions for redundancy removal with *k* sensors left, in which prosthetists can define *k* by themselves.

(1) Keep only one sensor in a cluster: To find the best single sensor of each cluster, we calculated PCC when comparing with the curve from selected sensors and the average curve from all sensors in one cluster.

For dataset *L*, there are three sensor selections in $C_{l_{a}}$ and seven sensor choices in $C_{l_{b}}$. We compute ten PCCs for each selection and repeat the calculation six times for six data frames. Table 1 gives the average PCC for all six data frames, in which $a_{0}$ to $a_{2}$ are from $C_{l_{a}}$, and $b_{0}$ to $b_{6}$ are from $C_{l_{b}}$. The red value means the best score of the metric among all choices.

The PCCs in cluster $C_{l_{a}}$ are all higher than 0.99, which shows the similarity to the centroid of this cluster. We choose sensor $a_{1}$ with highest PCC, which is 0.9997, to be the sensor that is kept. For cluster $C_{l_{b}}$, sensor $b_{3}$ with PCC of 0.9979 is the winner in PCC scores, so we choose $b_{3}$ to be kept.

The same result evaluation process is performed on the sensor strip *R*. For five sensors in $C_{r_{a}}$ and the other five sensors in $C_{r_{b}}$, $a_{2}$ and $b_{2}$ are selected after evaluating the PCC.

(2) Keep two sensors in a cluster: If the sensor designer chooses to leave two sensors for each cluster, we apply the same strategies to find the best two sensors. For $C_{l_{a}}$, there are three sensor selections and $C_{l_{a}}$ contains 21 sensor pairs.

**Figure 9:**
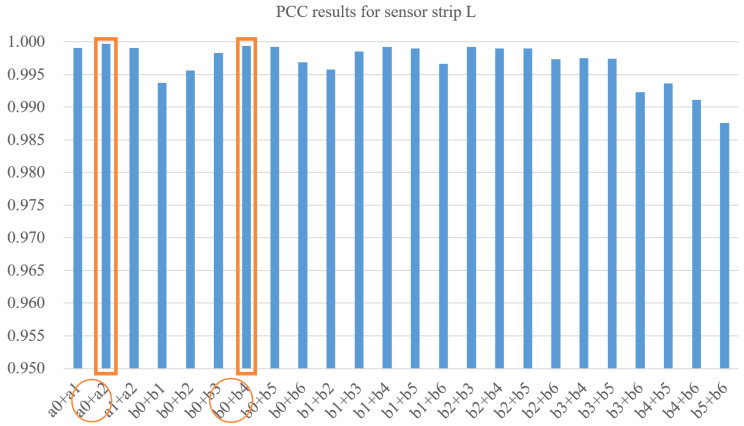
Results for selecting two sensors in dataset *L*. Orange circles indicate the best choices for each cluster.

Figure 9 shows the average results on six frames for all these 24 possible pairs. The best results for each cluster are marked. For $C_{l_{a}}$ we choose the combination of $a_{0}+a_{2}$, and for $C_{l_{b}}$ the pair of $b_{0}+b_{4}$ is kept at last. Similarly, we can choose $a_{0}+a_{3}$ for $C_{r_{a}}$ and $b_{1}+b_{3}$ for $C_{r_{b}}$ on the sensor strip *R*.

The high PCC provides evidence that the sensors’ data has high similarity. That means the pressure curves generated by selected sensors have high potential to replace the original layout of the sensors. The result of keeping one or two sensors are given as an example, while this approach could also suggest combinations with more sensors based on the opinion of the prosthetists.

### 5.4. Validation

To validate the redundancy removal result, we concatenate the data frames back into the whole dataset with 3000 points. Then, the pressure distribution of the whole test can be presented by the histogram of the probability density. Since the sensors have a reading range from 0 to 64 KPa, we set the number of bins to 64, and then calculate the JSD(P||Q). *P* is the pressure distribution of the corresponding cluster, which represents the initial sensor layout. *Q* is the pressure distribution of the selected sensors from the redundancy removal step.

**Figure 10:**
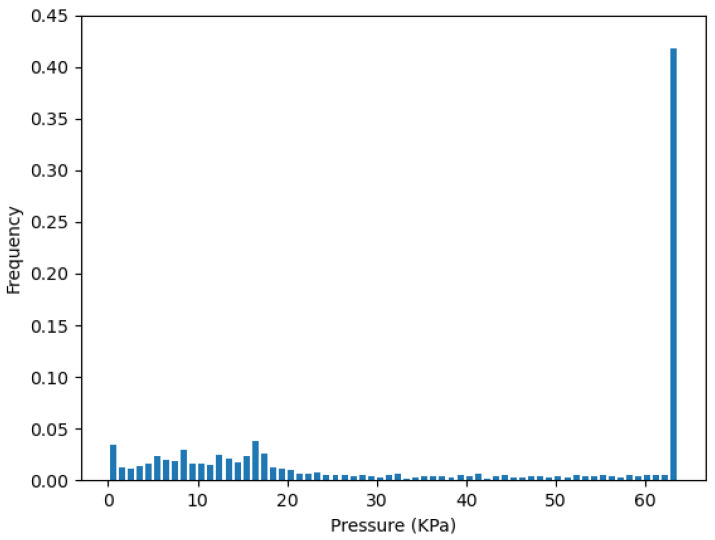
Pressure distribution from one-sensor $Rb_{2}$ in $C_{r_{b}}$.

**Figure 11:**
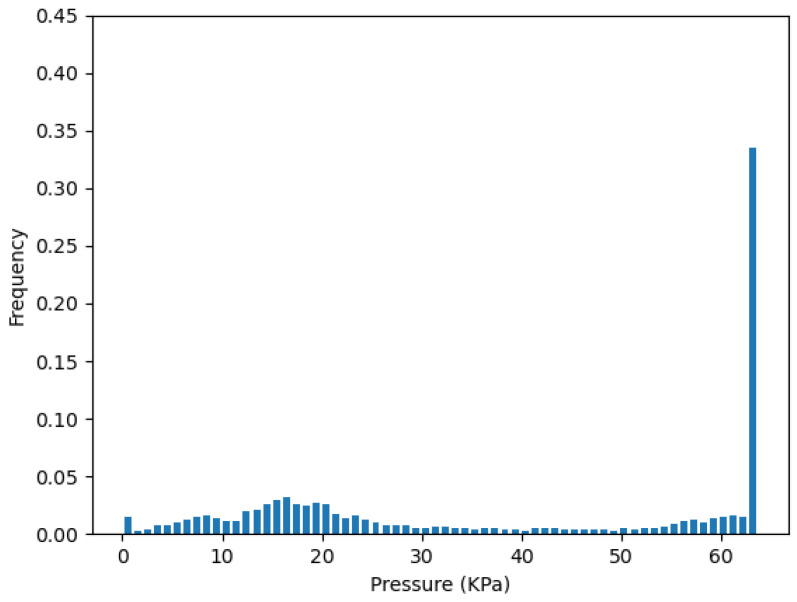
Pressure distribution from all sensors in $C_{r_{b}}$.

Figure 10 plots the pressure distribution from the one-sensor selection of the cluster $C_{r_{b}}$ on strip *R*, which is $Rb_{2}$. Figure 11 plots the pressure distribution of all sensors in cluster $C_{r_{b}}$. We can see they both have a high distribution on the saturation pressure of the sensor, which is 64 kPa.

**Figure 12:**
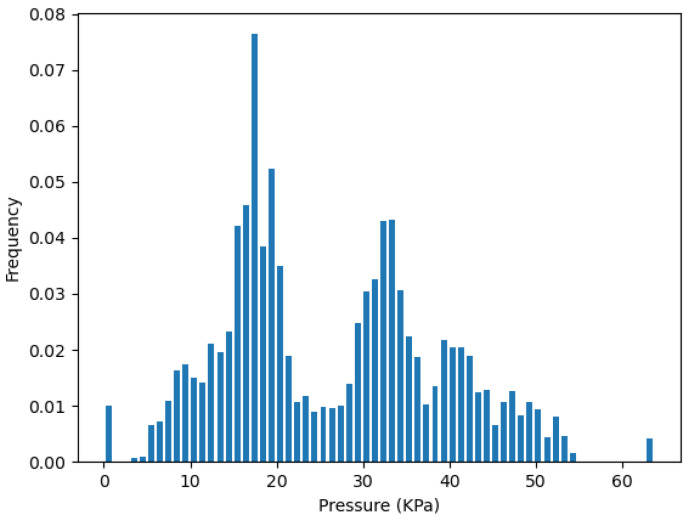
Pressure distribution from two-sensor $La_{0}$, $La_{2}$ in $C_{l_{a}}$.

**Figure 13:**
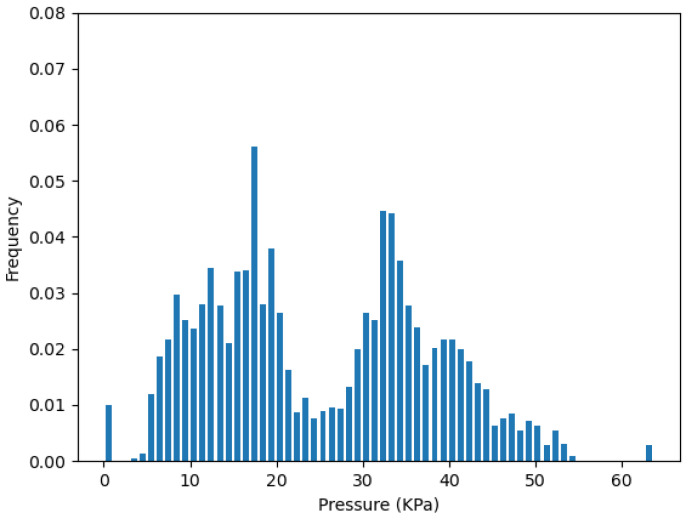
Pressure distribution from all sensors in $C_{l_{a}}$.

Similarly, Figure 12 plots the pressure distribution from the two-sensor selection of the cluster $C_{l_{a}}$ on strip *L*, which contains $La_{0}$ and $La_{2}$. It has a very similar distribution shape with the pressure distribution plot of all sensors in $C_{l_{a}}$ during our test, as shown in Figure 13.

Table 2 shows the JSD validation results of the selected sensor combinations after the redundancy removal procedure. Table 3 shows the validation results of the sensor selections with the minimum JSD values among all possible choices inside the corresponding clusters. The differences between these two tables are labeled in red. For the one-sensor selection, we can see that two of them are different from the suggested choice from the redundancy removal section, which are in $C_{l_{b}}$ and $C_{r_{a}}$. For the two-sensor selection, we find one different selection in $C_{r_{a}}$. The two differences that occur in cluster $C_{r_{a}}$ have an insignificant gap in JSD (<0.06), and the validation results are also relatively low (<0.1). That means the suggested sensor selections from the redundancy removal procedure are still credible in these two cases.

However, we observe a larger difference in the one-sensor selection in $C_{l_{b}}$, which indicates only selecting one sensor from the cluster $C_{l_{b}}$ is not an optimized solution. Since the two-sensor result is consistent in this cluster, it is better to keep two sensors rather than only selecting one.

**Figure 14:**
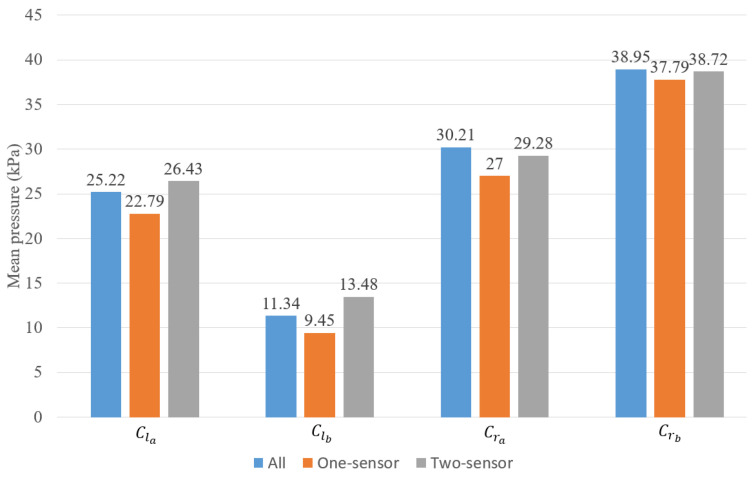
Validation of the mean pressure.

The mean pressure during walking is also useful for prosthetists [25] when analyzing the sensor data. Here, we will compare the average pressure of the four clusters from the redundancy detection model and the sensors after removal on the whole walking dataset. Figure 14 plots the mean pressure of the initial sensor deployment in each of the redundancy detection clusters and the selected sensor combinations from the sensor removal section. From this figure, we can see that the mean pressure of the two-sensor selection is very close to the original readings, with an average variation of 5% in all four clusters. $C_{l_{b}}$ has the highest variation of about 18.9%. For the one-sensor selection, the average variation is around 10%, and $C_{l_{b}}$ still has the highest difference of 16.7%. That means the prosthetist may require more sensors in area $C_{l_{b}}$ to obtain a more accurate mean pressure reading. In general, the two-sensor selection is better than the one-sensor selection for mean pressure measurement, which also meets our expectations.

Overall, our clustering-based analysis method can guide the removal of unessential sensors in the sensory system with high-density elements. With high PCC, the information on trends is proved to be kept. The JSD shows the high similarity of pressure distribution between the sensors to be kept, and the mean pressure validation shows satisfying results after sensor removal. These indicate that our clustering-based method to remove redundancy is effective.

## 6. Conclusions

We present a case study that aims to improve the sensor deployment for interfacial pressure measurement in the prosthetic socket by reducing the redundancy in local areas. In the paper, a clustering-based analysis method is proposed to evaluate the sensor density and give references to the removal of sensors. The SOM clustering algorithm is employed to build the redundancy detection models, and the K-means algorithm is used for cross-validation. Then, similarity metrics are used to guide the removal of unessential sensors with respect to the clustering result and help the sensor designers optimize the sensor configurations accordingly. A case study based on a clinical trial of an amputee during walking shows the effectiveness of our method. With ten sensors mounted on the posterior region, as well as the medial region inside the socket, their redundancy is detected by dividing the sensors into two clusters. Then, we remove the dispensable sensors evaluated by the PCC metric while keeping the main pressure features. The JSD evaluation result and the mean pressure comparison show the acceptable loss in the pressure features after redundancy removal, which validates the efficacy of our method. The proposed method can be useful in socket rectification and smart prosthetic socket design. It assists researchers in selecting efficient pressure sensor positions inside sockets. The reduced sensor deployment can still allow effective data acquisition in adjustable sockets, thus reducing the weight and complexity of the device.

Through the case study, we have detailed a method to reduce redundancy in a sensor layout scheme. To investigate further, this method can be applied to various patients and multiple sensor deployments. Currently, this is limited by our experimental conditions. We are also aware of the possible need to increase sensor density for higher coverage in some critical regions, which still need to be identified.

## Figures and Tables

**Table 1:** Results for selecting one sensor in dataset *L*. The red PCC scores indicate the winners of each cluster.

Sensor	Cluster	PCC
$a_{0}$	$C_{l_{a}}$	0.9986
$a_{1}$	$C_{l_{a}}$	0.9997
$a_{2}$	$C_{l_{a}}$	0.9919
$b_{0}$	$C_{l_{b}}$	0.9925
$b_{1}$	$C_{l_{b}}$	0.9950
$b_{2}$	$C_{l_{b}}$	0.9977
$b_{3}$	$C_{l_{b}}$	0.9979
$b_{4}$	$C_{l_{b}}$	0.9936
$b_{5}$	$C_{l_{b}}$	0.9922
$b_{6}$	$C_{l_{b}}$	0.9597

**Table 2:** JSD from the sensor removal results. The different choices between Table 2 and Table 3 are marked in red.

	$C_{l_{a}}$	$C_{l_{b}}$	$C_{r_{a}}$	$C_{r_{b}}$
1-sensor selection	$La_{1}$	$Lb_{3}$	$Ra_{2}$	$Rb_{2}$
JSD	0.084	0.224	0.092	0.045
2-sensor selection	$La_{0}$, $La_{2}$	$Lb_{0}$, $Lb_{4}$	$Ra_{0}$, $Ra_{3}$	$Rb_{1}$, $Rb_{4}$
JSD	0.018	0.036	0.017	0.010

**Table 3:** The sensor selection with the smallest JSD among all choices. The different choices between Table 2 and Table 3 are marked in red.

	$C_{l_{a}}$	$C_{l_{b}}$	$C_{r_{a}}$	$C_{r_{b}}$
1-sensor selection	$La_{1}$	$Lb_{4}$	$Ra_{3}$	$Rb_{2}$
JSD	0.084	0.134	0.039	0.045
2-sensor selection	$La_{0}$, $La_{2}$	$Lb_{0}$, $Lb_{4}$	$Ra_{1}$, $Ra_{2}$	$Rb_{1}$, $Rb_{4}$
JSD	0.018	0.036	0.009	0.010

## Data Availability

Not applicable.
